# Cross-Informant Compatibility of Depression Symptoms in Children: A Network Approach

**DOI:** 10.1007/s10578-022-01403-x

**Published:** 2022-08-02

**Authors:** Ayse Seneldir, Umit Akirmak, Sibel Halfon

**Affiliations:** 1 Department of Psychology, Istanbul Bilgi University, Istanbul, Turkey; 2 Vrije University Amsterdam, Amsterdam, Netherlands

**Keywords:** Cross-informant compatibility, Psychological networks, Depressive symptoms, Children

## Abstract

Utilizing multiple informants to assess children’s depressive symptoms increases diagnostic accuracy, reliability, and validity of inferences. However, previous studies have found low to moderate agreement among informants. We applied network statistics to gain insight into children and their mothers’ differential perceptions of depressive symptoms. The sample included children and mother dyads (*n* = 185) who applied to psychotherapy services at an outpatient university clinic. Mothers filled out the Child Behavior Checklist, which includes a depression subscale, and children filled out the Children’s Depression Inventory. We computed association networks for thirteen depressive symptoms separately for children and mothers using the graphical LASSO. *Sadness* had the highest strength centrality in the networks of both children and mothers, but the pattern of connectivity and centrality of other symptoms differed. We discussed our findings within the framework of network theory.

## Cross-informant compatibility of depression symptoms in children: A Network Approach

Multiple informants are the gold standard in child mental health assessments and ensure a more comprehensive and accurate evaluation of children’s problems [3, 4, 20]. However, there are often diverging views on children’s problems. Findings show that the consistency among the informants (i.e., cross-informant consistency) ranges from low to moderate in many studies [20]. This lack of consensus is especially true for problem behaviors that are more difficult to observe, such as internalizing problems (e.g., depression, anxiety, somatic concerns) where there is considerable disagreement about the presence and severity of child depressive symptoms [20]. Such inconsistencies complicate the creation of integrated treatment plans and pose the risk of not detecting depressive symptoms when data from only one source of information is collected, which may overshadow the need for intervention.

Currently, there is no reliable method for combining the perspectives of parents and children. Literature has generally relied on the degree of consistency and correlation coefficients as an index of the relationship between parent and child report assessments. When correlations agreement among informants is quantified by correlations, item-specific agreements or disagreements within an instrument are largely ignored. The present study addresses this issue by applying network statistics to assess item-specific cross-informant compatibility. While network statistics have been reliably applied to previous research on depression [18, 41], they have not been used as a tool to assess agreement among informants.

## Cross-informant consistency

In the field of child and adolescent mental health, clinicians typically collect data from multiple informants, such as caregivers, teachers, or peers [2, 21], which tends to show discrepancies. Various underlying reasons for these discrepancies were suggested in the literature: (i) cross-situational variability in children’s behaviors where different contexts and relationships may elicit different behaviors; (ii) different perspectives of informants, that is, the antecedents and consequences of children’s difficulties are perceived differently by children and parents; (iii) the visibility of children’s difficulties (i.e., internalizing vs. externalizing symptoms) with findings indicating that cross-informant agreement for externalizing problems are higher compared to the internalizing problems [6, 46, 59, 63]; and, finally, (iv) parent and child characteristics such as age, gender, and degree of psychopathology [15]. Furthermore, parents may also fail to recognize children’s emotional difficulties, leading to underreporting children’s symptoms [66].

Cross-informant compatibility of children’s problematic behaviors has been assessed with various scales, including Child Behavior Checklist (CBCL) [8], Youth Self-Report (YSR) [5], Child Depression Inventory (CDI) [53], and Teacher Report Form (TRF) [64]. Very few studies have applied these scales together to assess the depressive experiences of children from different informants. Yet, depression is a heterogeneous disorder and may include depressed mood or crankiness/anger in children, feelings of sadness and hopelessness, appetite changes, sleep problems, psychomotor agitation or retardation, feelings of guilt, and worthlessness, difficulty concentrating, and suicidal ideation [1]. Such heterogeneity suggests that many different combinations of depressive symptoms can be reported across informants [50]; however, these have not been assessed extensively. Most studies obtain measurements from parents using single scales that use sum scores, omitting the interpersonal and intrapersonal variability in the construct [27]. One study examined depression symptoms in children exposed to domestic violence by comparing the CBCL report and CDI reports [64]. The results showed that children were more likely than biological parents to report levels of maladjustment that varied depending on exposure to family violence. In another example, CDI and CBCL were used to diagnose children with depression in investigating motor activity levels [9], with children reporting most frequently anhedonia followed by negative mood. To the authors’ knowledge, no other study has used these two instruments to compare detailed differences and commonalities in aspects of children’s depressive experiences as reported by parents and children beyond more global assessments [48].

Typically, the degree of cross-informant compatibility is assessed using the Pearson correlation coefficient [7, 54, 58, 63, 67]. Other statistical methods such as the latent class approach [57], correlated trait method [33], and spearman rank correlation [53] were also utilized in assessing cross-informant compatibility. An overall scale score must be computed first by averaging scale items for each participant to compute correlation coefficients. Then, the agreement between informants’ scores can be computed. Correlation coefficients provide information about the rank-ordering of agreement levels between the two informants [38]. In addition to correlations, past research also examined the mean level differences among the informants’ reports. In this approach, the consistency between informants’ reports changes as a function of the standardized differences in mean scale scores [38]. The rank-ordering approach indexes the strength and direction of associations, and the mean difference approach indexes the magnitude of differences in the depressive symptoms. Nevertheless, both fail to detect the importance of specific symptoms (i.e., items) because correlations and means are calculated by summing or averaging over all items. The diagnostic value of an item is lost when it is averaged with the rest of the items on a scale. As an alternative, we propose to examine the contribution of individual items and their pattern of correlations to gain insights into the similarities and differences between informants’ ratings.

## Network Perspective on Depression

The network approach has recently gained attention in clinical psychology and psychiatry because it offers a new perspective on the conceptualization of mental disorders [28, 37] and is utilized as an alternative to the common factor model, especially in depression research [12]. Investigating psychopathology with the latent structure of disorders has been done in traditional methods. The latent dimensional or categorical disease models were found to be accurate in characterizing the non-psychiatric conditions; however, they seem to be limited in shedding light on the mechanism of mental disorders [13, 47]. This might lead to problems as long-searched-for latent causes do not exist that cause uncertainty in the formation of symptoms and frequency of covarying. The network approach supports that symptoms of mental disorders are “causing each other” in feedback loops. Also, when an individual symptom becomes more severe or elicited, this raises the likelihood that a connected symptom will occur, and even new episodes of disorder will appear [13]. According to the network approach, depressive experiences may not be caused by a single underlying cause. Instead, the complex causal interactions among the symptoms manifest themselves as the mental disorder, depression [26]. Specifically, the focus is on the specific symptoms and their pattern of association within this symptom network. For example, the activation of *being alone* can trigger *anhedonia*, and *anhedonia* can cause *fatigue* with all of these symptom interactions, eventually leading to a depressive episode (*loneliness* → *anhedonia* → *fatigue*) [13]. Thus, the complex and causal interactions of the symptoms lead to mental disorders [12].

An external event can trigger a symptom of depression (e.g., the loss of a loved one). This activation is likely to spread to the neighboring symptoms when a symptom is triggered. Once a larger portion of the network of symptoms is active, they can causally chain and activate each other even without an external event. Thus, even though external factors may trigger the symptoms, once they are triggered, they activate a network structure that maintains its activity through individual symptoms causing one another. According to the network approach, such a network structure of symptoms represents the dynamics of a mental disorder [12].

A network has two main components, nodes and edges [13]. Nodes represent the symptoms of a mental disorder, and edges represent the correlations between the symptoms [12]. In the network analysis, centrality is indicative of the significance of a symptom [14]. Typically, the centrality of a node is operationalized by the following: strength, closeness, and betweenness. Strength measures the sum of the direct connections to each node, while closeness is calculated by the sum of the distance from one node to other nodes in the network. Finally, betweenness quantifies how often a node is in the shortest path between two other nodes [21, 49]. Strength was found to be more reliable compared to betweenness and closeness [22, 36]. Network models inform us about the centrality of symptoms, overall association patterns in the symptom network, and how these characteristics change for different individuals.

Previous studies that utilized the network approach compared and contrasted the importance of depression symptoms and their associations. For example, *loneliness, sadness*, *pessimism*, and *self-hatred* were found to be the most central depression symptoms for adolescents, and the strongest association was between *sadness* and *crying* [46]. Another study showed that *loneliness*, *self-hatred*, *school dislike*, and *low self-esteem* were the most central symptoms, and the strongest connection was between *sadness* and *crying* in depressed children [42].

## Current study

This study was designed as naturalistic process-outcome research involving a standard battery of assessment scales administered to mothers and children. The mothers received the CBCL to measure the child’s problem behaviors and areas; however, the parent-reported CDI, which shares items with the CBCL, was not administered to the mothers to not overburden the participants and cause attrition. The children received the CDI because there is no child-report version of the CBCL. Even though CBCL is not a distinct measure of depression, it taps into DSM-focused depressive symptom items, has been used to measure depression in prior research, and CBCL scores from the Affective Problems scale have been found to closely correspond to DSM-IV major depressive disorder and dysthymia [23]. We utilized network statistics to study the cross-informant compatibility of children’s depressive symptoms based on the CBCL and CDI scales. Previous research utilized the CBCL and YSR scales in examining the cross-informant compatibility of depression symptoms [31, 38]. To the best of our knowledge, the consistency ratings of the CBCL and CDI have not been previously studied.

For this purpose, separate networks of some depressive symptoms were graphed based on the reports of children and mothers. For the first network, children’s reports of their depressive symptoms were utilized and based on CDI, a self-report depression scale for children. The second network was created by examining the mothers’ reports of children’s problems and was based on the CBCL scale. We expected to find differences in how the depressive symptoms are associated as a function of the informant (i.e., self-report vs. mother’s report). This approach also naturally indexes the differences in perception of depression symptoms as a function of the scale as the informants filled out different scales. Prior findings found low to moderate cross-informant consistency in depression; thus, we expected mothers and children to have different perceptions of depressive symptoms. We first explored the agreement of rank ordering of depressive symptoms with a correlation coefficient, but our primary interest was in the item-level agreements and disagreements. In addition, we examined whether the pattern of associations and centrality of depressive symptoms change as a function of the informant. In other words, assuming that children and mothers have different perceptions of the symptoms of depression, what they perceive as central to their experience is likely to be different as well. Finally, *sadness* and *crying* were expected to be central symptoms in our sample as they were found central in previous studies on adolescents and children.

## Method

### Participants

The data source for this study comes from Istanbul Bilgi University Psychotherapy Research Laboratory, which provides low-cost outpatient psychodynamic psychotherapy. Referrals were made by parents themselves or outside professionals. The mothers and the children were screened by a licensed doctoral-level clinical psychologist with over ten years of clinical experience and trained in developmental psychopathology and psychiatric interviewing techniques to determine whether the patients fit the study protocol inclusion criteria: ages between 4 and 10 years old, no psychotic symptoms, no severe developmental delays, no significant risk of suicide attempts or risk of harm to others and no drug abuse. The clinic is a university-based outpatient counseling center. When families apply for services, they go through a detailed intake process at the end of which the therapists write a narrative report and a psychosocial case formulation. Children do not receive a formal psychiatric diagnosis; however, therapists also assign a global function score to each child and fill out the Health of the Nations Scale for Children and Adolescents (clinician-rated HoNOSCA)[30] at the end of their assessment. In addition, the patients and their mothers are extensively informed before commencing therapy about research procedures. Mothers provide written informed consent, and the children provide oral assent concerning the use of their data, including questionnaires, videotapes, and transcripts for research purposes. This research was approved by Istanbul Bilgi University Ethics Committee.

The data used in this study is collected as part of a larger research program that aims to assess baseline predictors and effective treatment factors of outcome in psychodynamic child psychotherapy using a naturalistic process-outcome design^1^. All hypotheses and analyses are unique to the current research. The data was collected in routine clinical practice as part of the usual clinical assessment. There were no restrictions on patient selection outside our routine exclusion criteria, and the provision of care was treatment-as-usual without a control condition. The sample used in this study consisted of 187 children and their mothers who applied for services between October 2016–2020. Data from two dyads were not included in the statistical analyses because of many missing data in the CDI or CBCL measure. The analyses were conducted on 185 child-mother dyads, with no other missing data. All scales were completed by the mothers and the children. The age range of children was between 5 and 10 (*M* = 7.86, *SD* = 1.40).

The families were from low- to middle-income backgrounds in Istanbul, the largest metropolitan center in Turkey. Other sample characteristics are presented in Table 1. All the children who started psychotherapy at the clinic were new referrals and did not receive any prior treatment at our clinic. Most of them had long-standing problems. The sample mostly comprised of children who showed borderline or clinical levels of problem behaviors (Mean CBCL Total Problem score = 62), where scores over 60 indicate clinical functioning. According to their therapists, they had moderate impairment in psychosocial function on the Children’s Global Assessment Scale (CGAS)[61]. Moreover, according to therapists’ assessments on HoNOSCA item 9, 49.73% of children showed moderately severe anxiety and depression symptoms. Similar rates were reported by the mothers (around 40%); however, children’s self-report depressive experiences were markedly lower (around 20%; see Table 1).

**Table 1:** Demographic Characteristics of Children and Parents (n = 185)

Characteristics	Percent
Sex (children)	
Male	46.5
Female	55.6
Clinical Levels of Depression (CDI*)	
Male	22.1
Female	27.3
Clinical Levels of Depression (CBCL**)	
Male	41.41
Female	38.38
Severity of Anxiety and Depression (HoNOSCA)	
Moderately Severe	47.03
Severe to Very Severe	2.70
Socio-Economic Status	
Low	16.1
Low-Middle	25.6
Middle	43.9
Middle-High	12.8
High	1.7
Marital Status	
Married	83.6
Divorced	16.4
Mother’s Education	
No schooling	0.6
Elementary/Middle School	27.8
High School	30.4
University/Higher Education	41.2
Father’s Education	
No schooling	0.6
Elementary/Middle School	28.3
High School	32.3
University/Higher Education	38.7
Employed	
Father	92.0
Mother	45.9

Note. * CDI Clinical Depression Cut-off > 19, ** CBCL Clinical Depression Total score > 60

## Measures

### Children Behavior Checklist (CBCL)

The Child Behavior Checklist (CBCL) [5] is a widely used assessment tool that identifies problematic behaviors in children. We utilized the scale version that is appropriate for ages 6–18. Mothers are asked to respond to 112 items on a three-point rating scale (0 = *not true*, 1 = *somewhat or sometimes true*, and 2 = *very true or often true*). The items can be subdivided further into three categories: internalizing (e.g., depression, anxiety), externalizing (e.g., aggression, violence), and total problems. It has high internal consistency (CBCL 6–18: *α* = 0.97) and one-week test-retest reliability (CBCL 6–18: *r* = .94) [8]. In the current study, all three subscales showed good to high degrees of internal consistency (CBCL 6–18: *α* = 0.88 and *α* = 0.90, *α* = 0.94 respectively for internalizing, externalizing, and total score). For the 13 items included in the current analysis, the internal consistency was found *α* = 0.72.

### Children’s Depression Inventory (CDI)

The Children’s Depression Inventory (CDI) [43] is a self-report depression scale for children and adolescents. It consists of 27 items, each including three statements about a depression symptom scored on a three-point rating scale based on symptom severity (0 = *absence of symptoms*; 1 = *mild symptoms*; 2 = *severe symptoms*). The total score represents the severity of depressive symptoms. Participants can get 54 as the highest score, representing a severe level of depressive symptoms, and 0 as the lowest represents no presence of symptoms. The cut-off value is determined as 19; above indicates the presence of depression and below the absence of depression. CDI items are read by an examiner, and children are asked to choose one of the statements that best describe their experiences during the last two weeks. The original scale has good internal consistency (*α* = 0.82 to 0.89) [62]. We utilized the Turkish version of CDI [51], which has high internal consistency (0.81) and test-retest coefficient (0.80). In the current study, CDI total score showed good internal consistency (*α* = 0.83), and for the 13 items included in the current analysis, the internal consistency was found *α* = 0.63.

### Health of the Nation Outcome Scales child and adolescent Mental Health (HoNOSCA)

The HoNOSCA [30] includes clinical symptoms that the child may experience, such as depression, anxiety, behavioral, and severe psychiatric problems (eating disorder, psychosis, and severe substance abuse). It consists of 15 items, each rated from 0 (no problem) to 4 (severe impairment). The scale has good reliability and internal validity [30] and was adapted to Turkish [35]. Only item 9 (“problems with emotional and related symptoms”) was included in the current study to provide descriptive data on the extent to which children display depressive and anxiety symptoms (see Table 1).

### Data Analysis

Because the CDI and CBCL measures include overlapping and different items, we screened these scales to find the items that fall under the same depression symptom. We compared the 27 items of the CDI with those of the CBCL and identified 13 shared items (i.e., symptoms). Only these items were included in the statistical analysis (see appendix). Shared items are related to the following symptoms: *anhedonia, crying*, *disobedience*, *fatigue, loneliness*, *misbehavior*, *reduced appetite*, *sadness*, *schoolwork difficulty, self-blame, sleep disorder, unloved*, and *withdrawn*. To check whether some of these items are redundant and may be measuring the same underlying construct, we utilized the ‘goldbricker function’^2^ in the R package ‘networktools’[40]. The results indicated that none of the items in the CDI or CBCL networks were redundant. Thus, we included all 13 items^3^ in our statistical analyses.

Network analysis for both CBCL and CDI data was conducted with the JASP (version 0.16) [39] and R (version 4.2.0) [52] software. The relationships among the 13 symptoms of depression were explored by first building two association networks and examining the strength centrality of these symptoms separately for the shared CDI and CBCL symptoms. We examined the pattern of symptom associations and the strength centrality of these symptoms across CDI and CBCL networks through this method.

Symptoms were measured on an ordinal scale with three levels (0, 1, and 2), which were used as raw scores. We computed the “Graphical LASSO (‘’least absolute shrinkage and selection operator”) [29] with EBIC’’ method [22]. EBICglasso is used as an estimator since the networks have ordinal variables. The models were regularized Gaussian graphical models (GGMs) in which edges can be interpreted as ‘partial correlation coefficients’ [17]. Moreover, the sample size was small, thus, to have more consistent results bootstrapping was applied with an estimated 2500 samples. Non-parametric bootstrap was chosen since it tends to generate unbiased estimates with LASSO regularized edges [22, 37]. Centrality Stability analysis was provided to investigate the stability of the strength centrality index. The cut-off value for the centrality-stability coefficient (CS-coefficient) is determined as .25 for sufficient stability and .50 for good stability [22].

To estimate the cross-informant consistency, we also calculated the Spearman correlation between the total scores of the CDI’s and CBCL’s shared items. In addition, we examined whether socioeconomic status (SES), as reported by mothers, correlated with total scores of CDI and CBCL. Finally, we examined whether there are gender differences in the presence of depression symptoms. For this purpose, we conducted a Chi-Square test on the groups based on mean CDI cut-off scores (1: the presence of depression, 0: absence of depression) of boys and girls.

## Results

### Preliminary analyses

The Spearman correlation coefficient between the shared items of the CDI and CBCL measures was very weak and statistically non-significant, *r* = .06, *p* = .45, and *n* = 185. SES did not correlate reliably with CDI (*r* = − .03, *p* = .72, and *n* = 185) and CBCL (*r* = − .08, *p* = .27, and *n* = 185). There was no statistically significant difference between boys (*n* = 19, 22.1%) and girls (*n* = 27, 27.3%) in the presence of depression symptoms based on their CDI scores, *χ*^*2*^ (1, *N* = 185) = 0.66, *p* = .42.

### Network Analysis

**Fig. 1:**
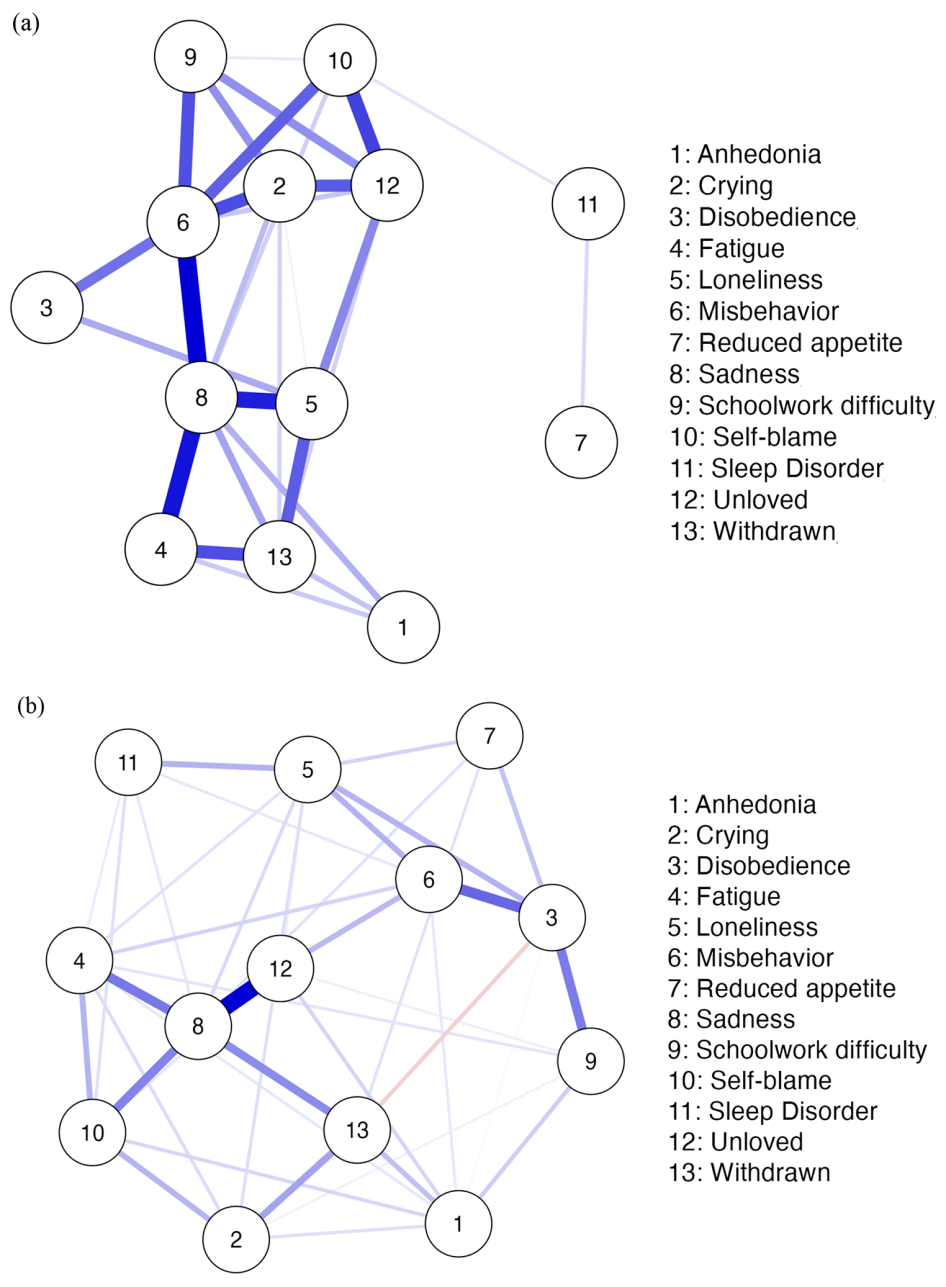
Network Models of Depression Symptoms. Figure 1a illustrates the depression symptom network based on children’s self-reports (CDI), and Fig. 1b illustrates the depression symptom network based on parent reports (CBCL). Note that the boldness of the lines implies the strength of association between the symptoms. Blue edges and red edges represent positive and negative relationships, respectively.

The regularized symptom networks of children and their mothers are presented in Fig. 1a (CDI) and Fig. 1b (CBCL). The results yielded sparse connections in both networks, but the sparsity was lower for CBCL (0.47) compared to CDI (0.64). In both networks, almost all symptoms were positively associated. A negative correlation was present only in the CBCL network, which was between *withdrawn* and *disobedience*.

The strongest association was between *sadness* and *unloved* in the CBCL network. The strongest association was between *sadness* and *misbehavior* in the CDI network, followed by *sadness* and *fatigue, and sadness* and *loneliness*. When Fig. 1a is examined, it can be seen that *sadness*, *misbehavior*, *loneliness*, and *crying* are at the center of the CDI network, while *reduced appetite* and *sleep disorder* are at the periphery and scarcely connected to the rest of the network. CBCL network displayed a different pattern of connections among the symptoms. The symptoms in the CBCL network appear to be more loosely connected and form two clusters. *Sadness* is in the center of the first cluster and is strongly connected to *unloved*, while *disobedience* is in the center of the second cluster and is strongly connected to *misbehavior* and *schoolwork difficulty*.

### Centrality analysis

**Fig. 2:**
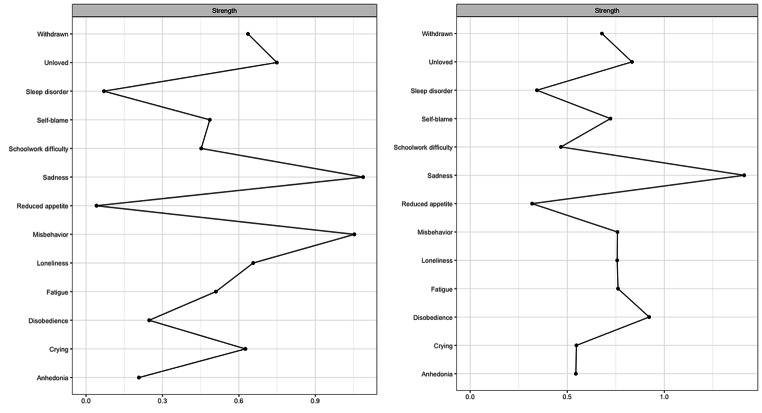
*Strength Centrality Estimates of the Network Models. Figure 2a presents the centrality analysis for the CDI network (children), and Fig. 2b presents the centrality analysis for the CBCL network (parents)*

Figure 2 presents the results of the strength centrality analysis. *Sadness* had the highest standardized strength centrality in the CBCL network (*z* = 2.53), followed by *disobedience* to a lower extent (*z* = 0.79). These central symptoms appear disjointed from each other and indirectly connected through other symptoms, most notably, *misbehavior* and *unloved*. *Sadness* had the highest strength centrality (*z* = 1.70) in the CDI network, closely followed by *misbehavior* (*z* = 1.60). In contrast to the CBCL network, these central symptoms were connected directly and appeared at the center of the CDI network. Additionally, *reduced appetite* has the least strength centrality in both CBCL (*z* = -1.34) and CDI (*z* = -1.46) networks.

The most central symptom in both networks was *sadness*, but the pattern of associations of *sadness* with the other symptoms varied. *Sadness* was more strongly associated with *unloved, fatigue*, *self-blame*, and *withdrawn* in the mothers’ network, while it was more strongly associated with *fatigue*, *loneliness*, and *misbehavior* in the children’s network. Thus, the perception of sadness and its close connections to other symptoms seemed to differ for mothers and children.

The stability of the centrality index was found to be sufficient ((CS(cor = 0.7) = 0.36) for both CDI and CBCL networks.

## Discussion

This study evaluated the consistency of depressive symptoms reported by children and their mothers using network statistics. Previous research has shown low agreement between parent and child reports of depressive symptoms [20], and the statistically non-significant correlation between children’s and mothers’ reports in our sample supported these findings. However, prior work mostly used correlational analyses to study the associations between parent and child report measures, whereas our network-modeling approach provided unique insights into how children and parents viewed children’s depressive symptoms. We computed mothers’ and children’s depressive symptom networks separately using children’s CDI and mothers’ CBCL ratings and examined the centrality as well as the pattern of associations of the depressive symptoms in the two networks. Our network graphs showed that the children’s most central nodes, based on strength centrality, were *sadness* and *misbehavior*, and, to a lesser extent, *loneliness, unloved, withdrawn*, and *crying*. In contrast, *sadness* and *disobedience*, and to a lesser extent, *unloved* were the most central nodes in the parent’s network. Children most strongly associated *sadness* with *fatigue*, *loneliness*, and *misbehavior*, while mothers most strongly associated *sadness* with *unloved*, *fatigue*, *self-blame*, and *withdrawn*. These results highlight the differential perception of depressive symptoms by mothers and children. Furthermore, present research contributes to the cross-cultural generalizability of the network approach by extending past work on the application of network statistics to symptom networks using different measures and a different cultural context.

Our results are partially consistent with [42], which is the only other study to use CDI in network analysis with a group of school-age Korean children and found loneliness, self-hatred, school dislike, and low self-esteem were the central symptoms of depression. *Loneliness* was also a central item in our sample but it was less pronounced compared to *sadness* and *misbehavior*. The centrality of *sadness* was distinct to our population. It is possible that because our sample was a clinical sample compared to [42], sadness was a pronounced experience, whereas their sample was a normal elementary school sample, who may be more prone to show interpersonal problems. We also found that symptoms related to proper conduct (*misbehavior* and *disobedience*) were central elements in children’s and mothers’ networks. In a previous study from a group of children from the same outpatient clinic, [34] also found that children with externalizing and co-occurring internalizing externalizing problems were concerned about acting-out and disrupting the household order. The concerns regarding disobedience/misbehavior and associated sadness may also be related to the cultural characteristics of our sample, where interpersonal harmony and acceptance are highly valued in Turkish families [25]. These children may be hypersensitive to disrupting the familial order and not complying with the parents’ expectations of proper conduct. This may be why crying was not one of the central items in the network. These children may hide overt behavioral expressions of their feelings due to emotional socialization practices in this culture. Studies have shown that individuals from collectivistic cultures have a tendency to exercise greater restraint over their emotions [45]. Moreover, it was found that parents more easily identify and name children’s sadness compared to other more aggressive emotions, which may be a threat to hierarchical relationship structures [16].

One further aim was to estimate cross-informant consistency using CDI and CBCL measures. The strength of association between depression symptoms shared by CDI and CBCL was weak and statistically non-significant. This finding may be related to the characteristics of our sample. The sample mostly comprised of parents with low socioeconomic status and parental education characteristics. More affluent and higher SES parents tend to have more knowledge about child development [11, 44, 56], and some studies, though limited, show that they make more accurate and elaborate attributions to children’s mental states [10, 43]. Moreover, the mothers’ network showed slightly higher connectivity (i.e., lower sparsity), but the connections were less strong, and the network was more widely spread out. Instead, there were slightly fewer connections (i.e., higher sparsity) in the children’s networks, but the connections were stronger. This suggests that for the children, their experience of depression centers around a few distinct symptoms. Instead, the mothers are able to recognize more symptoms yet have a hard time making the connections among these experiences; hence their network has less densely connected symptoms. In a previous study including parents from the same outpatient clinic, it was found that parents experienced difficulties making links between children’s behaviors and internal states. In other words, they displayed a low mentalization capacity [33]. This finding has important treatment implications. The parents would benefit from the guidance of the therapists in differentiating which symptoms are more pertinent for their children and making the necessary links among these experiences.

By itself, the weak correlation coefficient between the CDI and CBCL total scores does not allow for robust inferences. However, by applying network statistics and graphing the associations among the depression symptoms separately for mothers and children, we were able to show that both networks have certain commonalities. For example, *sadness* had the highest centrality in both children’s and mothers’ symptom networks. In the mothers’ network, sadness was strongly associated with *fatigue, self-blame, unloved*, and *withdrawn*, while in the children’s network, it was associated with *fatigue*, *loneliness*, and *misbehavior*. These associations suggest that children are focused on proper conduct, which, as stated before, is a central aspect of the Turkish harmonious family structure, based on obedience and conformity [24, 65]. Additionally, *sadness* was related to *fatigue* in both parties’ networks, but this association was weaker in the mothers’ network. Sadness was also associated with overt behavioral expressions of being withdrawn in the mothers’ network. These findings also have cultural implications. Somatic and behavioral symptoms are more prominent in cultures that do not encourage open emotional expression and reflection [45]. Somatic expressions of depression are more common than affective ones in Turkish adults [60]. However, this finding needs to be replicated with children. Although *sadness* is central in both networks, it also has different associations with the rest of the symptoms assessed within this study. Specifically, *sadness* was associated with *loneliness* in the children’s network, but this association was not evident in the mothers’ network, where *sadness* was strongly connected to *unloved*. These two perspectives are complementary and bring together a much more complex picture of children’s depression and sadness. Present findings have implications for treatment planning and interventions. It is important to assess both parents’ and children’s perspectives taking into account cultural considerations when assessing the experience of depression and not only focusing on overt signs of depression (i.e., disobedience and misbehavior) but also including evaluations of internal states that may not be readily visible from outside.

The network approach allows for inferences regarding the direction, nature, and significance of the associations among items of a scale [37]. Furthermore, it sheds light on the centrality of specific symptoms [49]. Therefore, it has the potential to inform researchers not only on the overall level of consistency but also on the specific points of similarities and differences in the pattern of symptom associations [13]. With the application of network statistics, we enhanced our understanding of the mechanisms underlying the differential perceptions of depression symptoms. The insights gained in this research would not have been achieved by the traditional assessment of cross-informant consistency through the rank-order and mean-differences approaches. In summary, network analysis is helpful in the assessment of children’s depression because it focuses on the item-level associations, which are disregarded in the traditional approaches that focus on the overall scale scores. Therefore, it will help create new measures or evaluate existing measures in item selection.

Our findings have practical implications. For example, some researchers recommend starting treatment for depression with the least central symptom in the network [55]. Knowing the centrality of the symptoms helps improve the way clinicians and therapists treat symptoms. Thus, practitioners and researchers can learn more about a child’s experiences of depression by applying network statistics to their data, especially when data from multiple sources are available. Moreover, it is possible that at the end of treatment, there would be an improvement between parents’ and children’s network compatibility and the level of connectivity within their networks as they work to form relationships between different aspects of their symptoms during psychotherapy. As such, network analysis could be used as a pre-post measure to assess changes in these indices. Finally, by integrating different perspectives, network analysis has the potential to shed light on how one’s symptoms may be affected by the cultural context within which they emerge. Future research can assess depression networks across different cultures to see which items are more connected depending on the cultural characteristics.

Our study has a few limitations. Overall, we had a small sample size, and the results should be interpreted with caution. However, network statistics were also applied in previous studies, and reliable results were obtained with a smaller sample size and a larger number of estimated parameters compared to our study [see 41]. The internal consistency for the CDI items included in the present study was found to be slightly lower than the recommended threshold. This might be due to including a small subset of the items that tap into different aspects of depressive symptoms. Furthermore, our focus was on the specific points of disagreement in the perception of depressive symptoms rather than broader generalizations of perceptions of these symptoms. For this reason, we believe that our inferences remain valid and our approach has theoretical and practical uses. Since the informants in our sample only consisted of mothers, it was not possible to examine mothers’ and fathers’ symptom networks and their relationship with children’s symptom networks separately. Comparing mother and father reports with reports for the children would provide further data about how parents differentially perceive children’s problems^4^. On a similar note, we were not able to include therapists’ assessment of children’s depressive symptoms which would be an important line of research in the future. The centrality stability was found somewhat reliable in our sample, but it was lower than the recommended threshold [22]. Thus, these findings should be interpreted with caution. Some discrepancies between the symptom networks of children and mothers may be attributed to the differences in the instruments.

We suggest that the child and parent versions of the CDI be used together in future studies to assess the impact of item wording and other differences from scales. Our research design was cross-sectional and not experimental, and thus causal inferences are not supported. Future studies can employ a longitudinal design with a larger sample size to index the course of change in the perception of depression symptoms. Such an approach is likely to provide stronger evidence for the causal links present among the symptoms of depression. Also, future studies can evaluate the generality of the approach presented in this paper by applying it to other measures of depression and clinical samples. Lastly, further researchers can focus on the comorbid mental disorders with depressive symptoms since the network approach allows for investigating the complex comorbidity, the covariance of symptoms, and bridging symptoms among multiple disorders [19].

## Summary

Collection of data from multiple sources (parents, peers, teachers, observers, etc.) is considered as best practice in assessing child psychopathology [2]. However, previous studies reported low to medium consistency across different raters. The present study outlines a network modeling-based approach that provides insight into these differences and thus contributes to the cross-informant compatibility literature. We compared children’s self-reports of depressive symptoms with those of mothers’ reports. Symptom networks were computed separately for children (CDI) and mothers (CBCL), and we examined the centrality of the depressive symptoms and the pattern of associations of the symptoms across these networks. When children’s ratings of depressive symptoms were examined, *sadness* and *misbehavior* appeared to be the most central nodes. Similarly, *sadness* and *disobedience* were the most central nodes according to mothers’ ratings. Using the network approach and focusing on the symptoms, we were able to demonstrate the commonalities between mother and child reports. There were also large discrepancies in the children’s and mothers’ depressive symptom networks. Children associated *sadness* with *fatigue*, *loneliness*, and *misbehavior*, while mothers associated *sadness* with *fatigue*, *self-blame*, *unloved*, and *withdrawn*. The existence of discrepancies between informant reports is not surprising and in line with prior research. This is why it is crucial in clinical assessments to use data from multiple informants and not solely rely on parent report assessments. Our findings have significant clinical implications regarding this issue and hope will guide treatment planning in the clinics. Network analysis has the potential to provide novel insights into assessing and understanding children’s symptoms of depression, especially when data from multiple sources are available.

## Data Availability

Raw data and data analysis files are available on the Open Science Framework site. https://osf.io/qj4n6/?view_only=652be8a493ba4bc38b671e43e9ed275e.

## Footnotes

1 Please see [32] for larger data collection procedures and other studies that emerged from this dataset.

2 We thank the Reviewers for this suggestion.

3 Please see the supplementary materials for a list of CDI and CBCL items used in the present research.

4 We thank the Reviewers for this suggestion.
